# Dr. Denman on the Structure of Cancerous Parts

**Published:** 1812-05

**Authors:** Thomas Denman

**Affiliations:** Mount-street


*To the Editors of the Medical and Physical Journal.*

GENTLEMEN,

I SHALL feel much obliged by your inserting the following notice in your very useful publication.

In a small tract on the subject of cancer, not long ago published, I gave, on the authority of Dr. Baillie, Mr. Home, Mr. Abernethy, and Mr. Brodie, a description of the structure of a cancerous part in its primordial state; from which, after a certain time, there was always found to proceed an order of filaments which were supposed peculiar to, and diagnostic signs of, cancer. It was further suggested and endeavored to be proved, that the spreading of cancer from one part to another, and in various directions, was not made by the absorption of any newly generated or perverted humor, endued with any specific properties, but by the creeping of these filaments to other parts; and that the different discharges from cancerous parts were not to be considered as primary causes of the disease, but as consequences of its continued, varied, or increased, action. Yet it cannot be doubted but that, in the progress of cancer, the whole frame may be so contaminated by the disease and its general effects, as ultimately to overwhelm all the powers of the constitution by corruption, by exhaustion, or by the destruction of parts necessary for the purposes of life.

It has, however, been surmised or doubted whether such or similar filaments are not found after the extirpation of every kind of tumor, at least of many of them, when there was no reason to suspect the existence of cancer, or apprehension of it at any future time. This point I hope some of your numerous and well-informed correspondents will be able to decide, and, if they think it of as much importance as I do, they will lose no opportunity of making their observations upon it. Should it be proved that such filaments do generally exist in tumors, we may inquire whether there be any, and what, difference between the filaments existing in parts affected with different diseases, as to their constituent parts, structure, and arrangement: and, while this investigation is carrying on, opportunities may be afforded to detect that state of parts which disposes to, or makes them capable of, producing cancer. This disease is so terrible, and so frequent, that it is most desirable to discover or devise some means by which it may be prevented or cured, and an accurate history of it will probably in an eminent degree contribute to those ends.

I remain, Gentlemen,

*Mount-street,* Your very humble Servant,

*March* 12, 1812. THOMAS DENMAN.

